# Hemoxygenase-1 Promotes Head and Neck Cancer Cell Viability

**DOI:** 10.3390/antiox11102077

**Published:** 2022-10-21

**Authors:** Marilina Mascaró, Exequiel G. Alonso, Karen Schweitzer, Martín E. Rabassa, Jessica A. Carballido, Agustina Ibarra, Eliana N. Alonso, Vicente Bermúdez, Lucía Fernández Chavez, Georgina P. Coló, María Julia Ferronato, Pamela Pichel, Sergio Recio, Valentina Clemente, Maria Eugenia Fermento, María Marta Facchinetti, Alejandro C. Curino

**Affiliations:** 1 Laboratorio de Biología del Cáncer, Instituto de Investigaciones Bioquímicas de Bahía Blanca (INIBIBB), Universidad Nacional del Sur (UNS)-CONICET, Dpto. de Biología, Bioquímica y Farmacia (UNS), Bahía Blanca 8000, Argentina; 2 Centro de Investigaciones Inmunológicas Básicas y Aplicadas (CINIBA), Facultad de Ciencias Médicas, Universidad Nacional de La Plata (UNLP), La Plata 1900, Argentina; 3 Instituto de Ciencias e Ingeniería de la Computación (ICIC), Universidad Nacional del Sur (UNS)-CONICET, Bahía Blanca 8000, Argentina; 4 Unidad de Cirugía de Cabeza y Cuello, Hospital Municipal de Agudos “Dr. L. Lucero”, Bahía Blanca 8000, Argentina

**Keywords:** hemoxygenase-1, nucleus, cancer, head and neck

## Abstract

Head and neck squamous cell carcinoma (HNSCC) is a remarkably heterogeneous disease with around 50% mortality, a fact that has prompted researchers to try new approaches to improve patient survival. Hemoxygenase-1 (HO-1) is the rate-limiting step for heme degradation into carbon monoxide, free iron and biliverdin. We have previously reported that HO-1 protein is upregulated in human HNSCC samples and that it is localized in the cytoplasmic and nuclear compartments; additionally, we have demonstrated that HO-1 nuclear localization is associated with malignant progression. In this work, by using pharmacological and genetic experimental approaches, we begin to elucidate the mechanisms through which HO-1 plays a role in HNSCC. We found that high HO-1 mRNA was associated with decreased patient survival in early stages of HNSCC. In vitro experiments have shown that full-length HO-1 localizes in the cytoplasm, and that, depending on its enzymatic activity, it increases cell viability and promotes cell cycle progression. Instead, HO-1 does not alter migration capacity. Furthermore, we show that C-terminal truncated HO-1 localizes into the nucleus, increases cell viability and promotes cell cycle progression. In conclusion, we herein demonstrate that HO-1 displays protumor activities in HNSCC that depend, at least in part, on the nuclear localization of HO-1.

## 1. Introduction

Hemoxygenase-1 (HO-1) is an inducible 32 kDa isoform of HO that belongs to the phase II detoxifying enzymes. HO-1 catalyzes the first rate-limiting step in heme degradation, leading to the formation of equimolar quantities of carbon monoxide (CO), biliverdin and free iron, thus acting as an antioxidant defense system [1,2].

It has been demonstrated that the HO-1 protein, as well as its three byproducts, play an important role not only in physiological processes, but also in other diseases, cancer being one of them [3]. Heavy metals, inflammation, UV radiation and ROS, which are factors frequently associated with the initiation and progression of cancer, induce HO-1 levels.

HO-1 has been shown to display anti-inflammatory and antioxidant properties, which suggest protection to the host. Moreover, this antitumoral role has been demonstrated in some tumors from solid tissues such as colon [4], liver [5], prostate [6], head and neck [7], breast [8] and lung tumors [9]. However, a protumoral role has also been reported for most tumor types [3], including some tumors mentioned above such as head and neck [10] and lung cancer [11]. All these reports together suggest that HO-1 may play a dual role in cancer, and that this role may depend on the type of tissue and/or on the protein subcellular location. Interestingly, although it has been originally described as a microsomal enzyme, its subcellular location is dynamic, and nuclear HO-1 expression has been further reported in many tumor tissues [3].

Head and neck squamous cell carcinoma (HNSCC) is the sixth most common cancer in the world, and despite advances in the last 30 years in the understanding of this disease as well as in improvements in diagnosis and treatment options, the 5-year survival rate remains around 50% [12,13]. The main risk factors for HNSCC are tobacco and alcohol exposure as well as HPV infection. In the last years, the advancement in deep sequencing studies led researchers to gain insight into the particular molecular landscape underlying the etiology of HNSCC [14,15]. These studies have contributed to explaining the heterogeneity of tumor behavior and treatment outcomes.

We have previously demonstrated that HO-1 is upregulated in human tissues of HNSCC and that nuclear location of HO-1 is associated with tumor progression [10]. However, its role and the influence of HO-1 subcellular localization on tumor progression have not been studied in detail.

The aim of this work is to study the relevance of HO-1 expression, its subcellular location and, thus, its enzymatic activity in HNSCC. Our results show that HO-1 mRNA expression is associated with shorter overall survival at earlier stages of the disease. By pharmacological and genetic approaches, we show that the overexpression of HO-1 in HN13 cell line increases cellular viability by promoting cell cycle progression, and that it depends on its enzymatic activity. We also show that the overexpression of a truncated HO-1, which is localized into the nucleus, increases cell viability. Furthermore, by pharmacological modulation of HO-1 in a human mixed primary cell culture of SCC, we confirm the above results, suggesting that HO-1 promotes HNSCC malignancy.

## 2. Materials and Methods

### 2.1. Bioinformatic Analysis

We used RNA-seq TCGA-HNSC data from the GDC Data Portal of NIH to perform the bioinformatic analysis. The Cancer Genome Atlas profiled 528 head and neck squamous cell carcinomas (HNSCCs) to provide a comprehensive landscape of somatic genomic alterations. The data were downloaded using the Xena Browser (https://xenabrowser.net/datapages/, accessed on 29 October 2021). Both counts and samples were filtered according to different criteria. Regarding samples, Basaloid squamous cell carcinoma, spindle cell, Lip and Metastatic were removed since our study required those samples to be discarded. After the merge of the count matrix and samples, the final configuration that was used for the analysis consisted of 530 samples and 60,488 genes. Overall, 486 samples correspond to the primary tumor and 44 correspond to normal solid tissue. R studio was used to carry out the study. To analyze the differential expression of HO-1 mRNA expression, the necessary tests were previously carried out to check the assumptions: equal variances in the groups (tumor and normal) and normality in the distribution of the data. To determine equality of variances, we used the Levene test with an alpha of 0.05. To determine normality, we used the Kolmogorov–Smirnov normality test. Finally, we verified the differential expression of HO-1 mRNA with the Welch Two Sample t-test, also calculating the effect size with the Cohen d index. Finally, clinical data were considered, and statistical tests were performed to study the correlation between the HO-1 mRNA expression value and different variables, such as tumor location and disease stages.

### 2.2. Cell Culture

Human HNSCC cell line HN13 as well as human immortalized keratinocyte cell line HaCaT (both kindly provided by Dr Silvio Gutkind, UCSD, San Diego, CA, USA), were routinely maintained in Dulbecco’s modified Eagle’s medium (DMEM; Invitrogen) supplemented with 10% fetal bovine serum (Natocor, Villa Carlos Paz, Argentina) and an antibiotic-antimycotic solution (#15240062, Gibco) at 37 °C in a humidified 5% CO_2_ air atmosphere [10].

### 2.3. Human Tumor Tissue and Cell Culture

A tissue sample of surgically excised squamous cell carcinoma (SCC) of the epidermis (UNS-SCC-01) was obtained from a patient from the Hospital Municipal de Bahía Blanca, Bahía Blanca, Argentina with written consent and institutional review board approval.

The UNS-SCC-01 tumor sample was processed mechanically and enzymatically. After the tissue was minced by scalpel blade into 1 mm^3^ pieces, an incubation step using 0.1% collagenase type I at 37 °C for 40 min while the sample was shaken vigorously to separate cancer cells was performed. Then, cells were centrifuged at 300× *g* for 5 min, and the supernatant was discard. Afterwards, the incubation step was repeated nine times. After the final incubation, cells were cultured in DMEM/F12 (Invitrogen) supplemented with 10% (*v*/*v*) fetal bovine serum (FBS, Natocor), penicillin (100 U/mL, Gibco) and streptomycin (100 μg/mL, Gibco) at 37 °C in a humidified 5% CO_2_ air atmosphere.

### 2.4. Pharmacological Modulation of HO-1

HN13 and HaCaT cells as well as a human mixed primary cell culture were treated with 20–80 uM hemin [8], an activator of HO-1, or its vehicle for 24–72 h. Additionally, cell lines were treated with 1–10 uM Zinc Protoporphyrin (ZnPP) [16], an inhibitor of HO-1 enzymatic activity, or its vehicle for 24–72 h. The human mixed primary cell culture was treated with 10 uM Tin Protoporphyrin IX (SnPP) [16], an inhibitor of HO-1 enzymatic activity, or its vehicle for 24–72 h.

### 2.5. Stable Overexpression of HO-1 in Human HN13 Cell Line

HN13 cells were plated in 35 mm dishes until 70% confluent, and the transfection procedure was performed the following day using Lipoafectamine (Invitrogen) according to the manufacturer’s instructions. To stably overexpress the native full length form of HO-1, the expression plasmids FLAG-FL-HO1 and FLAG-FL-control (kindly donated by Dr. L.Y, Chau, Taipei Medical University, Taiwan) were used, and stable transfectants (HN13-FL-HO-1 and HN13-CTRL) were selected by incubating cells with hygromycin (1 mg/mL) [17]. To evaluate the relevance of activity and subcellular location of HO-1, FLAG-FL-HO-1 (H25A) and FLAG-t-HO-1 expression plasmids (both kindly donated by Dr. L.Y, Chau, Taipei Medical University, Taiwan), respectively, were used, and the stable transfectants (HN13-FL-HO-1(H25A) and HN13-t-HO-1) were selected by incubating cells with Hygromycin (1 mg/mL) [17]. Finally, overexpression and subcellular location of HO-1 expression was evaluated by direct immunofluorescence of FLAG.

### 2.6. Evaluation of Cell Viability and Proliferation

HN13 and HaCaT cells were seeded into 96-multi-well dishes in DMEM 10% FBS at 37 °C in a 5% CO_2_ atmosphere. After treatment with hemin, ZnPP or its vehicles, the medium was removed, and adherent cells were fixed with 100% methanol. After washing, cell viability was evaluated by crystal violet assay as described previously [18]. Data obtained (OD per well) were expressed as percentage of control. The experiment was carried out at least three times in triplicate. Additionally, at selected time points, cell counting was performed as described previously [18].

### 2.7. Immunofluorescence

To evaluate HO-1 expression by indirect immunofluorescence, cells were cultured on coverslips, and IIF was carried out as previously described, with a few modifications [8,19]. Then, cells were incubated with a rabbit polyclonal anti-HO-1 (SPA-896; Stressgen Bioreagents) followed by an Alexa Fluor 488-conjugated secondary Ab (Molecular Probes). To evaluate FLAG expression in stably transfectants cells by direct immunofluorescence, cells were incubated with a mouse monoclonal anti-FLAG M2-FITC (F4049; Sigma Aldrich, Burlington, MA, USA) following the manufacturer’s instructions. Finally, cells were stained with DAPI and mounted on Mowiol^®^ 4-88 (#81381, Sigma Aldrich). Cells were analyzed by using Nikon Eclipse E600 fluorescence microscope or Leica TCS SP2 confocal microscope.

### 2.8. Cell Cycle Analysis

Cell cycle analysis was performed as described previously [8]. HN13 cells treated with hemin, ZnPP or their vehicles, or stable transfectant HN13 cells, were incubated at selected time points. Then the cells were trypsinized and washed with ice-cold PBS. After that, the cells were fixed with ice-cold 70% ethanol and stored at −20 °C until one month. Then, the cells were stained with 50 μg/mL Propidium Iodide (PI, Roche) plus RNase A (100 µg/mL) in PBS and analyzed for DNA content by FACScan flow cytometry (Becton Dickinson, Franklin Lakes, NJ, USA). At least 100,000 cells per sample were evaluated, and data were analyzed using Cell Quest Software (Becton Dickinson).

### 2.9. Wound-Healing Assay

As was previously described [19], cells were seeded in 35 mm dishes until confluence. A monolayer was cut with a sterile pipette tip, and cells were incubated with 80 μM hemin, 10 μM ZnPP or their respective vehicles for 8 h. Photographs were taken at 0 and 8 h at the same position. Images were captured with an inverted microscope (Nikon Eclipse TE2000-S) equipped with a digital camera (Nikon Coolpix S4). Results were expressed as percentage of the wound area calculated as: (100% × % areat = 8 h)/% areat = 0 h. T-scratch software (version 1.0) was used to calculate acellular area [20].

### 2.10. Immunoblotting

HN13 and HaCaT cells were treated with 80 μM Hemin, 10 μM ZnPP or their respective vehicles for 24 to 72 h, both at 37 °C in a 5% CO_2_ atmosphere. Human mixed primary cell culture was treated with 80 μM Hemin, 10 μM SnPP or their respective vehicles for 48 h at the same conditions previously mentioned. Then, cells were lysed with hypotonic buffer. After centrifugation, an equal amount of protein was resolved by SDS-polyacrylamide gel electrophoresis and transferred onto a PVDF membrane (Osmonics Inc., Gloucester, MA, USA). The membrane was blocked and incubated with specific antibodies to HO-1, cyclin D1, p27 and actin, overnight at 4 °C, followed by incubation with horseradish peroxidase-labelled secondary antibody for 2 h at 37 °C. The detection system and densitometric analysis were performed as described previously [8,19].

### 2.11. Immunohistochemical Staining

Immunohistochemical staining was performed as previously described [8]. In brief, after deparaffination, blocking endogenous peroxidase activity and non-specific binding sites steps were carried out. Slides were then incubated with primary rabbit polyclonal anti-HO-1 antibody (dilution 1:100; SPA-896; Stressgen Bioreagents) overnight at 4 °C followed by incubation with diluted biotinylated secondary antibody and then incubation with VECTASTAIN ABC Reagent (Vector Laboratories, Inc., Newark, CA, USA). For negative controls, the primary antibody was omitted. Diaminobenzidine/H_2_O_2_ was used as a substrate for the immunoperoxidase reaction. Finally, they were lightly counterstained with hematoxylin and mounted with Permount (Fisher Scientific, Pittsburgh, PA, USA) for analysis by bright-field microscopy.

### 2.12. Statistical Analysis

Statistical significance between more than two groups was evaluated by two way-ANOVA, and means ± standard error (SEM) were compared with the Bonferroni’s Test (cell viability, wound healing and cell cycle assays). When two groups were analyzed, the statistical significance between groups was evaluated by one way-ANOVA, and the means ± standard error (SEM) were compared with the Dunnett’s test (wound healing and proliferation assays). Statistical significance was determined at *p* < 0.05 level. Analysis was performed using the GraphPad Prism software package, version 5.00 (Graph Pad, San Diego, CA, USA).

## 3. Results

### 3.1. HO-1 mRNA Expression Is Associated with Patient Survival and HPV (-) Infection

**Figure 1:**
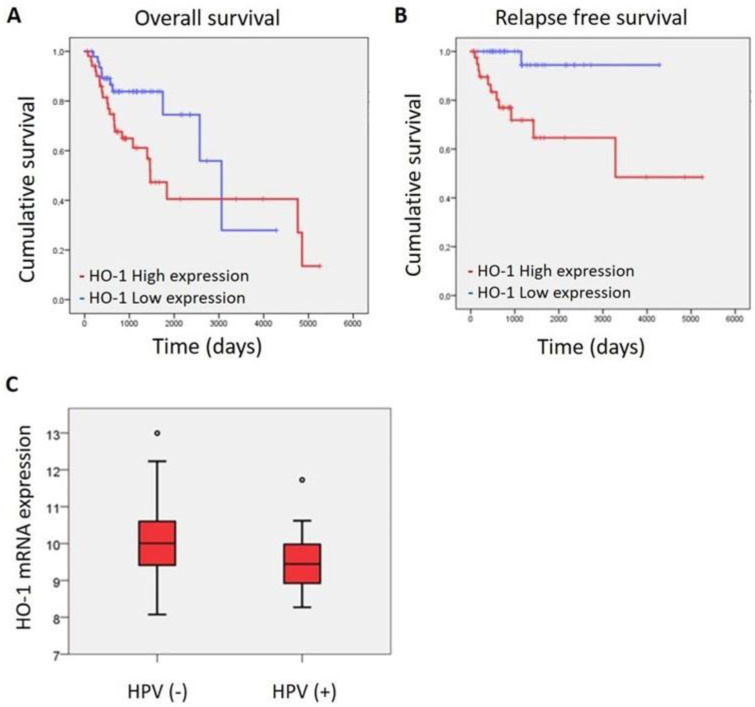
HO-1 mRNA expression is associated with overall survival, relapse-free survival and risk factor HPV status. (**A**) Kaplan–Meier overall survival curves for HNSCC patients stratified as Stage 1 + 2 according to low and high HO-1 expression level (*n* = 217, Breslow test, *p* = 0.046). (**B**) Kaplan–Meier relapse-free survival curves for HNSCC patients stratified as Stage1 + 2 according to low and high HO-1 mRNA expression level (*n* = 217, Log Rank test, *p* = 0.003). (**C**) Comparison of HO-1 mRNA levels in HNSCC according to HPV status (*n* = 88, ANOVA test, *p* = 0.034).

We have previously demonstrated that HO-1 protein expression is associated with malignant progression in human samples of HNSCC [10]. Now, taking advantage of the molecular landscape analysis and clinical information of HNSCC tumors provided by public TCGA data [14], we set out to find out whether HO-1 mRNA expression is associated with survival. We found that HO-1 mRNA expression is associated with overall survival and relapse-free survival depending on stage disease. At early stages (I–II), a high level of HO-1 mRNA expression is significantly associated with worst overall survival (*p* < 0.046; Figure 1A) and relapse-free survival (*p* < 0.003, Figure 1B), while, at late stages (III–IV), the significant association between HO-1 mRNA expression and survival is lost.

Moreover, taking into account that HPV status infection as well as tobacco and/or alcohol consumption are the main risk factors for HNSCC disease and that HNSCC etiology determines the subtype of HNSCC with a particular biology and a concomitant response to treatment [13,14], we asked ourselves if there could be any association between such risk factors and HO-1 mRNA expression. Interestingly, we found that HNSCC-HPV (-) showed a higher expression of HO-1 mRNA than HNSCC-HPV (+) (*p* < 0.0034) (Figure 1C), whereas tobacco and/or alcohol consumption failed to show association (Supplementary Figure S1).

These results suggest that HO-1 would be a bad prognosis factor, at least at earlier stages of the disease, and that HO-1 may play a role in HNSCC.

### 3.2. Pharmacological HO-1 Activation Increases Cell Viability and Cell Cycle Progression of HN13 Cells

Taking into account the association of HO-1 expression with tumor progression and survival, we evaluated whether HO-1 may play a role in HNSCC biology. To that end, we used the human normal keratinocyte cell line, HaCaT, and the human HNSCC tumor cell line, HN13, to evaluate the effect of the pharmacological activation of HO-1 with hemin on cell viability, proliferation and cell cycle progression.

**Figure 2:**
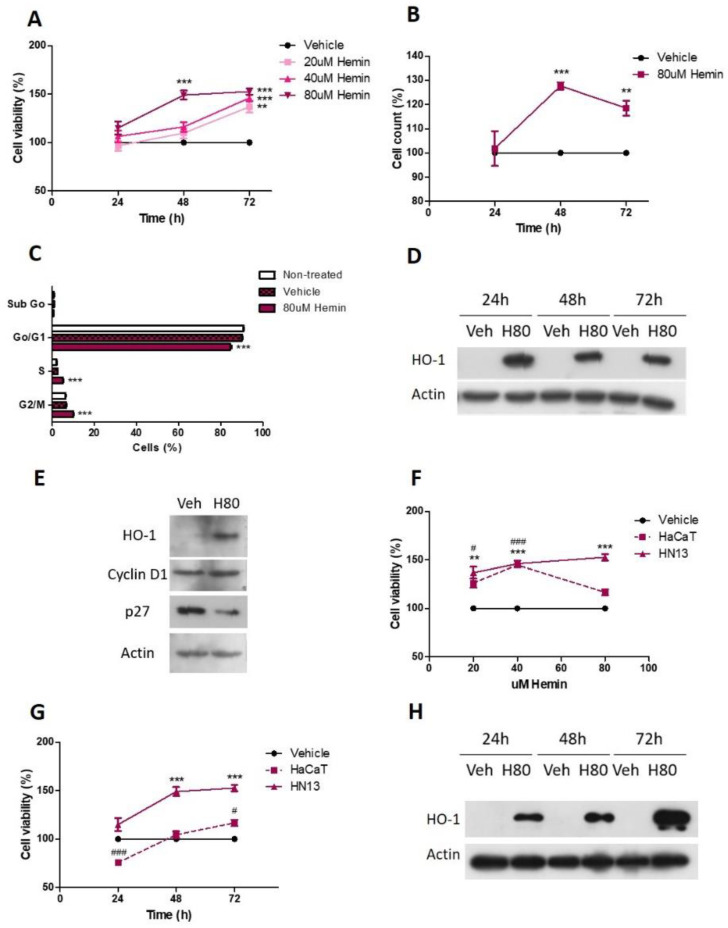
Pharmacological activation of HO-1 promotes cellular viability and cell cycle progression of HN13 cells. (**A**) HN13 cell viability was evaluated after treatment with hemin (20–80 uM) or its vehicle for 24 h to 72 h. (**B**) HN13 cell viability was evaluated after treatment with 80 uM hemin or its vehicle for 24 h to 72 h. (**C**) HN13 cell cycle progression and (**D**) HO-1 protein levels in HN13 cell line were evaluated after treatment with 80 uM hemin (H80) or vehicle (Veh) for 24 h to 72 h. (**E**) Protein levels of cyclin D and p27 after HO-1 activation with 80 uM hemin in HN13 cell line. (**F**) HaCaT cell viability was evaluated after treatment with hemin in dose-response assay at 72 h (**G**) and time-response assay using a 80 uM concentration, comparing the effects with those exerted in HN13. (**H**) HO-1 protein levels in HaCaT cell line were evaluated after treatment with 80 uM hemin (H80) or vehicle (Veh) for 24 h to 72 h. The results are expressed as cell viability percentage = (hemin group × 100/vehicle group). ** *p* < 0.01 and *** *p* < 0.001 with respect to the vehicle in HN13 cells. # *p* < 0.05 and ### *p* < 0.001 with respect to the vehicle in HaCaT cells.

We found that HN13 cell viability increases after 80 uM hemin treatment at 48 h (149.1 ± 4.56, *p* < 0.001) and 72 h (152.7 ± 3.23, *p* < 0.001), as assessed by crystal violet assay (Figure 2A). Lower hemin concentration (20 and 40 uM) increases HN13 cell viability at 72 h (20 uM: 136.9 ± 6.08, *p* < 0.01 and 40 uM: 146.1 ± 3.73, *p* < 0.001; Figure 2A). The effect of 80 uM hemin treatment at 48 and 72 h was confirmed by manual cell count (Figure 2B). In addition, we analyzed cell cycle progression and found that 80 uM hemin decreases cell count in the Go/G1 phase (*p* < 0.001) and increases cell population in the S (*p* < 0.001) and G2/M (*p* < 0.001) phases compared to its vehicle (Figure 2C), thus showing that HO-1 pharmacological activation induces cell cycle progression. As expected, we found that 80 uM hemin increases HO-1 protein levels (Figure 2D), demonstrating that hemin is an inductor of the HO-1 expression in this cell line. Moreover, we found that 80 uM hemin increases cyclin D1 and decreases p27 protein levels, thus explaining the mechanisms through which HO-1 stimulates HNSCC cell cycle progression (Figure 2E).

In order to analyze whether there were differences in the response to HO-1 activation between tumor and non-malignant cells, we also studied the pharmacological activation of HO-1 in the non-malignant keratinocyte-derived HaCaT cell line. Importantly, we found that HaCaT cell viability increases following hemin treatment, although very slightly and to a lesser extent than HN13 cells, both at 72 h (Figure 2F) and using 80 uM hemin (Figure 2G). As expected, hemin also increases HO-1 protein expression in this non-malignant cell line (Figure 2H).

### 3.3. Pharmacological HO-1 Inhibition Decreases Cell Viability

Taking into account the above results where pharmacological HO-1 activation enhances HO-1 protein expression promoting cell proliferation, we evaluated if the antitumor effects depend on HO-1 byproducts. For this purpose, we inhibited HO-1 enzymatic activity with the HO-1 inhibitor, zinc protoporphyrin (ZnPP), and evaluated its effects on cell viability, proliferation and cell cycle progression.

**Figure 3:**
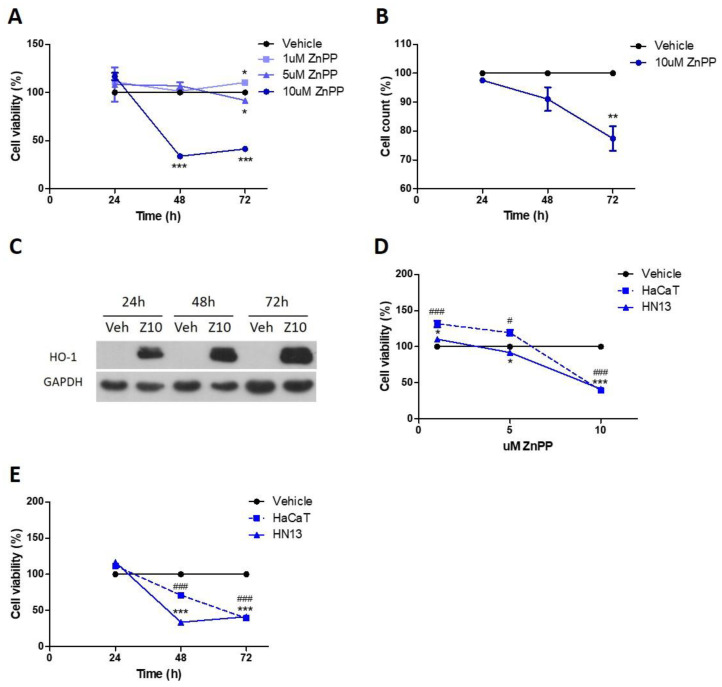
Pharmacological inhibition of HO-1 decreases cell viability of HN13 cells. (**A**) HN13 cell viability was evaluated after treatment with ZnPP (1, 5 and 10 uM) or its vehicle for 24, 48 and 72 h by crystal violet assay. (**B**) HN13 cell viability was evaluated after treatment with 10 uM ZnPP or its vehicle for 24, 48 and 72 h by manual cell count. (**C**) HO-1 protein expression was evaluated after treatment with 10 uM ZnPP or vehicle for 24 h to 72 h in HN13 cell line. HaCaT cell viability was evaluated after treatment with (**D**) ZnPP in dose-response assay at 72 h and (**E**) time-response assay using 10 uM concentration, comparing the effects with HN13 cells. The results are expressed as cell viability percentage = (ZnPP group x 100 / vehicle group). * *p* < 0.05, ** *p* < 0.01 and *** *p* < 0.001 with respect to the vehicle in HN13 cells. # *p* < 0.05 and ### *p* < 0.001 with respect to the vehicle in HaCaT cells.

We found that ZnPP inhibits cell viability in a concentration-dependent manner, as assessed by crystal violet staining. At 72 h, ZnPP decreases cell viability (5 uM: 91.86 ± 2.33, *p* < 0.05 and 10 uM: 41.44 ± 0.59, *p* < 0.001; Figure 3A). Then, we confirmed this inhibition of 10 uM SnPP treatment at 72 h by manual cell counting (Figure 3B), obtaining similar results. We then analyzed HO-1 protein levels following 10 uM ZnPP treatment at several time points. As expected, we found that ZnPP greatly increases HO-1 protein levels at all times studied (Figure 3C).

We further compared the effect of pharmacological inhibition of HO-1 by ZnPP in the HaCaT cell line. We found that HaCaT cell viability decreases after 10 uM ZnPP treatment at 48 and 72 h (Figure 3D,E), although to a lesser extent than in HN13 cells.

### 3.4. Pharmacological Modulation of HO-1 Does Not Alter Migratory Capacity

**Figure 4:**
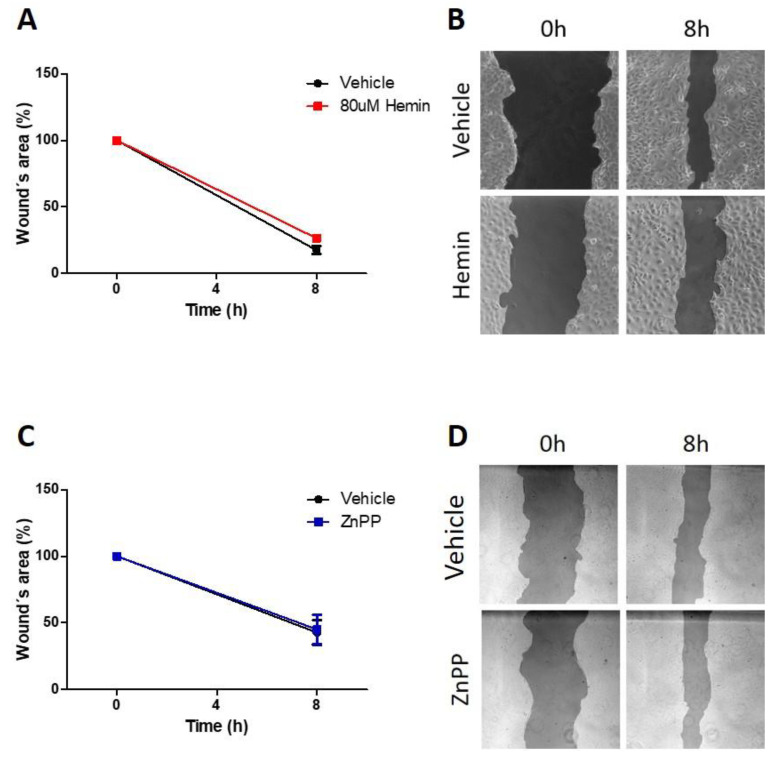
Pharmacological modulation of HO-1 activity does not alter HN13 cell migration. (**A**) HN13 cell migration was evaluated after treatment with 80 uM hemin or its vehicle for 8 h. (**B**) Representative images of an acellular area in 80 uM hemin-treated and vehicle-treated conditions at 0 and 8 h from (**A**) are shown. (**C**) HN13 cell migration was evaluated after treatment with 10 uM ZnPP or its vehicle for 8 h. (**D**) Representative images of an acellular area in 10 uM ZnPP treated and control conditions at 0 and 8 h from (**C**) are shown.

In order to evaluate whether HO-1 is involved in the migratory capacity of HN13, we treated these cells with hemin or ZnPP and their respective vehicles. We found that activation of HO-1 by 80 uM hemin (Figure 4A,B) or inhibition of HO-1 activity by 10 uM ZnPP (Figure 4C,D) did not alter cell migration, as assessed by wound-healing assay.

### 3.5. Nuclear HO-1 Expression Is Enhanced after Hemin Treatment, Whereas Strong Cytoplasmic HO-1 Is Found following ZnPP Treatment

**Figure 5:**
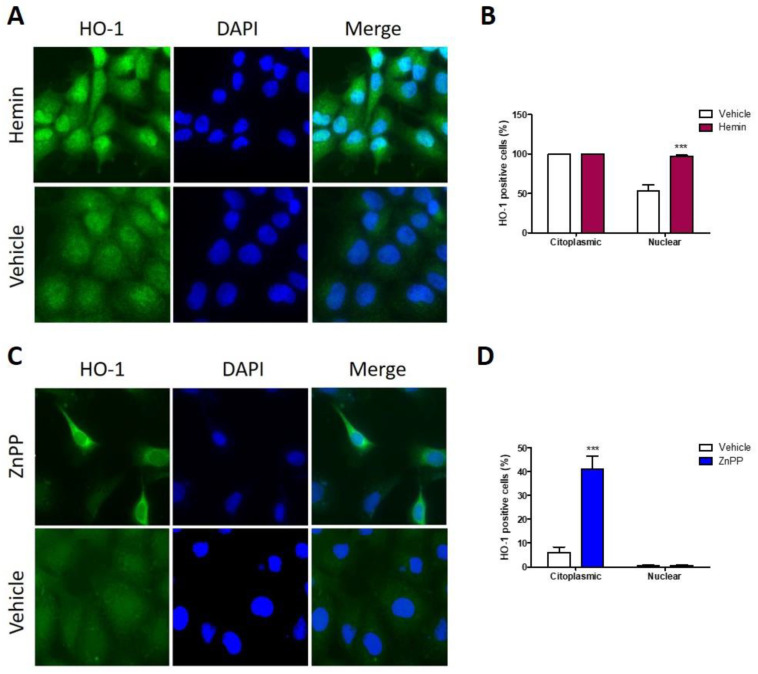
HO-1 subcellular localization in HN13 cells following pharmacological modulation. Representative images of HO-1 expression in HN13 cells after treatment with (**A**) 80 uM hemin or its vehicle for 24 h. (**B**) Semi-quantification of nuclear HO-1-expressing and cytoplasmic HO-1-expressing cells after hemin treatment. (**C**) Representative images of HO-1 localization in HN13 cells after treatment with 10 uM ZnPP or its vehicle for 24 h. DAPI was used to stain nucleus. (**D**) Semi-quantification of nuclear HO-1-expressing and cytoplasmic HO-1-expressing cells after ZnPP treatment. *** *p* < 0.001 respect to vehicle.

We have previously demonstrated that HO-1 nuclear localization associates with poorly differentiated tumors in HNSCC, as studied when using human samples [10]. As we have shown above, HO-1 hemin activation increases HO-1 protein expression and stimulates cell viability of HN13 cells. Therefore, we evaluated if hemin activation of HO-1 is able to increase HO-1 cytoplasmic to nuclear translocation. We found that hemin treatment increases HO-1 nuclear localization (Figure 5A,B). In addition, HO-1 inhibition by ZnPP increases HO-1 protein expression but decreases cell viability. Therefore, we evaluated if HO-1 enzymatic inhibition by ZnPP affects HO-1 nuclear localization. Indeed, ZnPP treatment augmented HO-1 expression but, contrary to hemin effects, it retained HO-1 into the cytoplasmic compartment (Figure 5C).

### 3.6. Nuclear HO-1 Increases Cell Viability

**Figure 6:**
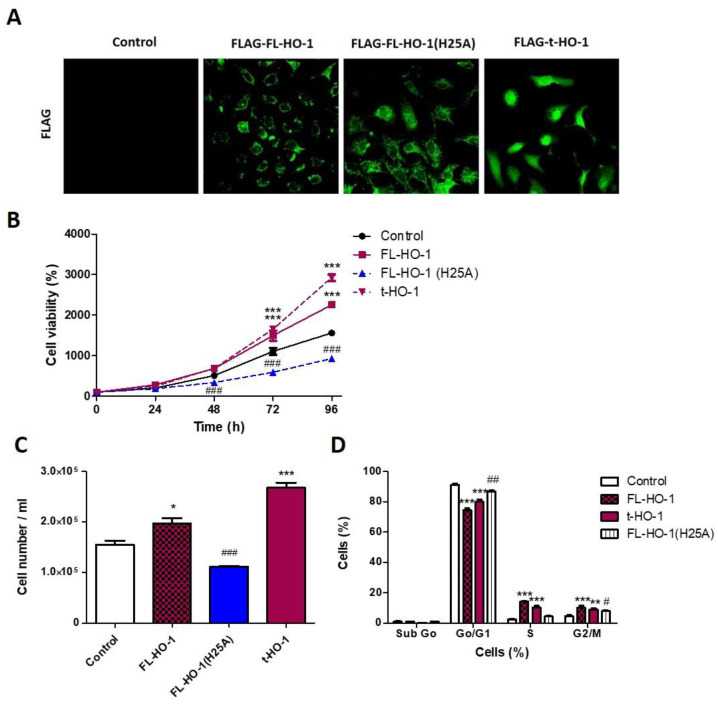
Subcellular localization and loss of enzymatic activity of HO-1 affects HN13 cell viability. HN13 cells were transfected with FLAG vectors bearing FL-HO-1, FL-HO-1 (H25A) or t-HO-1 constructs. (**A**) Representative images of direct immunofluorescence for FLAG showing differential subcellular location of HO-1 expression among stable transfectants HN13 cells. (**B**) Cell viability of the stably transfected HN13 cells was evaluated at 24 through 96 h. (**C**) Cell number of the stably transfected HN13 cells was counted at 72 h. (**D**) Cell cycle progression of the stably transfected HN13 cells was evaluated at 96 h. The results are expressed as cell viability percentage = (selected time group × 100/time 0 h group), being selected time 24 h to 96 h. * *p* < 0.05, ** *p* < 0.01 and *** *p* < 0.001 with respect to the control. # *p* < 0.05, ## *p* < 0.01 and ### *p* < 0.001 respect to FL-HO-1 (H25A).

Considering that ZnPP as well as hemin are able to produce HO-1-independent biological effects [21], and that hemin treatment does not allow one to discriminate between cytoplasmic and nuclear HO-1-expressing cell populations, we generated HN13 cells stably overexpressing the enzymatically active full length HO-1 (FL-HO-1), enzymatically inactive full length HO-1 (FL-HO-1-H25A) or truncated HO-1 (t-HO-1). We found that HN13 FL-HO-1 and FL-HO-1-H25A cell lines overexpress HO-1 into the cytoplasmic compartment, but the HN13 t-HO-1 cell line overexpresses HO-1 mostly into the nuclear compartment (Figure 6A). Then, we studied the effects of such genetic modifications on HN13 cell viability and proliferation as well as cell cycle progression.

The overexpression of FL-HO-1 increases cell viability (Figure 6B) and proliferation (Figure 6C) as well as cell cycle progression (Figure 6D). On the contrary, the overexpression of enzymatically inactive FL-HO-1 (H25A) decreases all the cellular processes previously mentioned (Figure 6A–C), suggesting that HO-1 byproducts play a role in the promotion of cellular proliferation. In addition, we also found that overexpression of t-HO-1, which localizes into the nucleus, increases cell viability and proliferation (Figure 6A–C), showing that nuclear localization of HO-1 also promotes HNSCC progression.

### 3.7. Pharmacological Modulation of HO-1 Affects Squamous Cell Carcinoma Cells Viability

**Figure 7:**
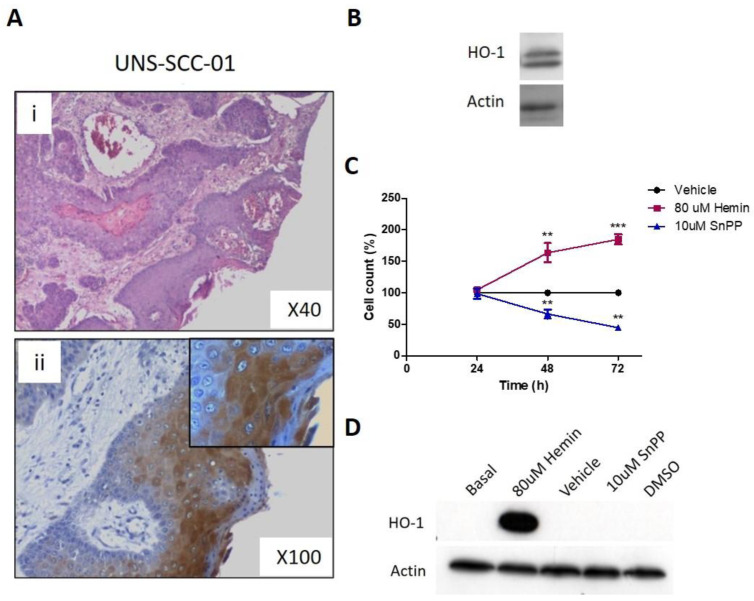
Expression and pharmacological modulation of HO-1 in human mixed primary cell culture of squamous cell carcinoma. (**A**) Representative images of hematoxylin eosin staining (**i**) and HO-1 staining (**ii**) by immunohistochemistry a tumor biopsy of human SCC. (**B**) HO-1 expression of sample shown in (**A**) by immunoblot. (**C**) Cell viability of the human mixed primary cell culture after treatment with 80 uM hemin or 10 uM SnPP or their respective vehicles at 24 to 72 h. (**D**) HO-1 protein expression evaluated after treatment with 80 uM hemin or 10 uM SnPP or their respective vehicles at 48 h. The results are expressed as cell viability percentage = (selected time group × 100/time 0 h group), with a selected time from 24 h to 96 h. ** *p* < 0.01 and *** *p* < 0.001 with respect to the vehicle.

Taking into account the obtained results using HN13 cell line, we further evaluated the effect of the pharmacological modulation of HO-1 using hemin and SnPP in a mixed primary cell culture obtained from a patient’s squamous cell carcinoma biopsy tissue. By immunohistochemistry, we show that HO-1 is expressed by tumor cells in the cytoplasmic and nuclear compartments (Figure 7A). The treatment of the primary cell culture with 80 uM hemin or 10 uM SnPP and their respective vehicles at 24 to 72 h shows that hemin increases cell viability, whereas SnPP decreases it at 48 and 72 h (Figure 7C). We also found that hemin treatment induces HO-1 overexpression, whereas SnPP fails to affect HO-1 expression (Figure 7D).

## 4. Discussion

We have previously demonstrated by using human tissue samples that HO-1 is expressed in the cytoplasmic and nuclear compartments of tumor cells in head and neck squamous cell carcinoma (HNSCC) and that nuclear HO-1 is associated with a worse prognosis of patients [10]. We have also reported that HO-1 mRNA and protein expression are higher in tumor tissues compared to normal counterparts. Altogether, these results suggest that HO-1 may be associated with HNSCC tumor progression. However, whether HO-1 plays a causal role in HNSCC has, as far as we know, been understudied.

In this work, we demonstrated that the enzymatic activity as well as the subcellular location of HO-1, play a crucial role in the behavior of HNSCC cells, by using pharmacological and genetic approaches. In addition, we performed bioinformatic studies on TCGA gene expression datasets.

We demonstrated that high HO-1 mRNA expression is associated with a worse survival in the earlier stages of HNSCC. Moreover, although we showed that HO-1 mRNA expression is higher in HPV (-) risk factor population, which is a HNSCC subtype with limited treatment options [13], whether an association does exist between HO-1 mRNA expression and survival in this population remains unknown because of the limited number of cases in the analyzed database. On the contrary, although a protective role of HO-1 against nicotine cytotoxicity in oral cancer cells and immortalized keratinocytes has been demonstrated [22], we failed to find an association between HO-1 mRNA expression and tobacco or alcohol risk factors.

Recently, others authors have also analyzed the TCGA database and reported that HO-1 mRNA expression is associated with a favorable prognosis [23]. However, they also reported that HO-1 mRNA levels are not significantly different between normal and tumor samples. Taking into account the heterogeneity of HNSCC, it is possible that the selection made by different researchers of same parameters, whether tumor type, site of tumor or tumor stage, may explain the differences obtained.

In this work, we demonstrated a protumor activity of HO-1 on biological processes, such as cell viability and cell cycle progression, in HN13 cells after pharmacological activation by hemin as well as after genetic overexpression of the enzymatically active full-length HO-1 form. Moreover, we demonstrated that such protumor activity of HO-1 depends on its enzymatic activity, since we demonstrated the reversal of the protumor activity when we inhibited it pharmacologically using ZnPP, and when we genetically overexpressed the enzymatically inactive mutant H25A full-length HO-1. Of note, while HO-1 activation enhances cytoplasmic-nuclear translocation, when we inhibit HO-1, it is retained in the cytoplasmic compartment, thus reflecting the relevance of the combination of expression, activity and location in the role of HO-1.

Our results also suggest that the human normal keratinocyte cells, HaCaT, are less sensitive to pharmacological modulation of HO-1 than tumor cells even though HaCaT showed a higher HO-1 expression than HN13. Even so, it has been demonstrated that the HO-1/AMPK axis induced by hemin contributes to ROS scavenging and protects keratinocytes to UVB damage, showing a beneficial effect of HO-1 in normal keratinocytes [24].

Recent reports have stated that HO-1 may be implicated in the antitumor effect of natural compounds extracted from plants [25,26,27,28,29] and curcumin analogs [23,30] in HNSCC cells. These studies mostly reported that HO-1 is involved in the apoptotic cell death of primary and metastatic oral cancer cells through p38 signaling [23,25,26,30], although ERK1/2 and Akt signaling were also implicated [27]. On the contrary, other authors reported that HO-1 rescued oral cancer cells from oxidative stress-induced apoptosis by TW-37 after HO-1 genetic overexpression [31] and by cadmium after hemin treatment and genetic HO-1 silencing [32]. It is well known that HO-1 may be induced by different stimuli as oxidative stress, hypoxia and oxidative injury, among others. In this work, we activated HO-1 by hemin and further confirmed such effects by genetically manipulating HO-1 expression, enzymatic activity and localization. We hypothesize that our target strategies fail to trigger the above signaling pathways or trigger a different one, showing the protumor potential of HO-1 in this type of tumor and opening the way to evaluate what other stimulus may be promoting this HO-1 role in HNSCC. Of note, Shi et al. demonstrated that high and moderate mRNA HO-1 levels are associated with lower tumor regression after radiotherapy in human nasopharyngeal cancer biopsies [33]. Furthermore, Lv et al. demonstrated that HO-1 play a role in the chemoresistance to cisplatin treatment in laryngeal cancer cells [34].

In our research, we also demonstrated that pharmacological modulation of HO-1 failed to alter the migratory capacity of HN13 cells, suggesting that HO-1 may be relevant mainly for the primary tumor growth. The role of HO-1 in the migratory and invasion capacity of HNSCC cells was poorly studied, even despite the use of oral metastatic cancer cells to evaluate potential treatments for HNSCC. For example, it was reported that HO-1 is involved in the migration, invasion and vasculogenic mimicry impairment of oral cancer cells after oxysophocarpine treatment, which decreases HO-1 expression [29]. Other authors reported that low concentrations of Withaferin A (WFA) inhibit the migratory and invasive capacity of oral cancer cells depending on ROS. However, HO-1 expression was stimulated by WFA treatment, but HO-1 implication was not fully studied [35].

In our previous publication [10], we showed that nuclear HO-1 expression correlated with higher histological grades. In addition, we reported an increase in nuclear expression of HO-1 in a murine model of squamous cell carcinoma carcinogenesis where cytoplasmic HO-1 was expressed in pre-neoplastic lesions and nuclear HO-1 was expressed in tumor tissues [10]. In the present work, we confirm that the pharmacological activation of HO-1 by hemin increases HO-1 nuclear expression in HN13 cells. However, pharmacological treatment with hemin does not allow us to discriminate between HN13 cell populations overexpressing HO-1 in either the cytoplasm or nucleus to evaluate the relevance of the subcellular compartment on the protumor activity of HO-1. By overexpressing the truncated HO-1 form, which translocates into the nucleus and loses its enzymatic activity (as was reported by Hsu et al.), we demonstrated that truncated HO-1 promotes HN13 cell viability. A nuclear HO-1 protumor role has been proposed for others tumor types as lung SCC, NSCLC, prostate, breast, colon, gliomas and some hematological malignancies [3]. To our knowledge, this is the first report showing the functional relevance of the nuclear HO-1 on biological processes, such as cell viability and cell cycle progression, in HNSCC.

Regarding the mechanisms underlying HO-1 nuclear translocation in HNSCC, several enzymes are able to cleave HO-1 as cathepsin B, calpain-1, calpain-2 and signal peptide peptidase (SPP). Its participation was demonstrated in hematological malignancies [36,37] and lung cancer [17]. In HNSCC, although cathepsin B [38] and calpain-1 [39] were reported to be overexpressed, and their expression has been identified as an independent unfavorable prognostic factor, their role in HO-1 truncation in HNSCC cells remains to be demonstrated.

Further research should be conducted to investigate under what tumor microenvironmental stimuli HO-1 translocate into the nucleus, and its relation with other capabilities and enabling characteristics, thus contributing to HNSCC aggressiveness.

Finally, by using a mixed primary cell culture from a human squamous cell carcinoma, which recapitulates the real tumor microenvironment, we further support the protumor activity of HO-1 observed using the HN13 cell line. We demonstrated that HO-1 promotes cell viability and that this depends on its enzymatic activity.

In summary, by using pharmacological and genetic approaches, we demonstrated that HO-1 plays a protumor role and that both the enzymatic activity and the subcellular location of HO-1 determine its participation in HNSCC malignancy.

## 5. Conclusions

In conclusion, HO-1 mRNA expression is associated with shorter overall survival at the earlier stages of HNSCC. The overexpression of HO-1 in the HN13 cell line increases cellular viability by promoting cell cycle progression, and this depends on its enzymatic activity. Additionally, HO-1 does not alter the migratory capacity of HN13 cells when modulated pharmacologically. Interestingly, the overexpression of truncated HO-1, which is localized into the nucleus, increases cell viability. Furthermore, the above findings were confirmed by pharmacological modulation of HO-1 in a human mixed primary cell culture of SCC. These findings suggest that the combination of expression, activity and location of HO-1 plays a significant role in HNSCC malignancy.

## Data Availability

Data are contained in the article and the Supplementary Materials.

## Supplementary Materials

The following supporting information can be downloaded at: https://www.mdpi.com/article/10.3390/antiox11102077/s1, Figure S1: HO-1 mRNA expression and risk factors, tobacco and alcohol.
