# Supplementary figures and images for: Hemoxygenase-1 Promotes Head and Neck Cancer Cell Viability
Source: Antioxidants (Basel). 2022 Oct 21;11(10):2077. doi: 10.3390/antiox11102077 (PMC9598840; doi:10.3390/antiox11102077)

**A**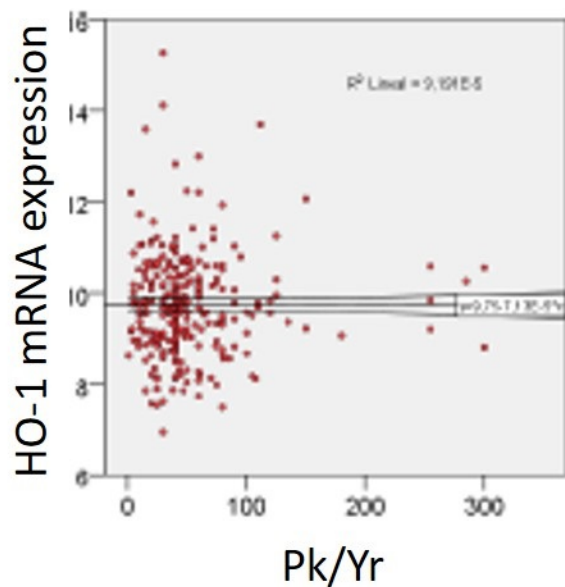**B**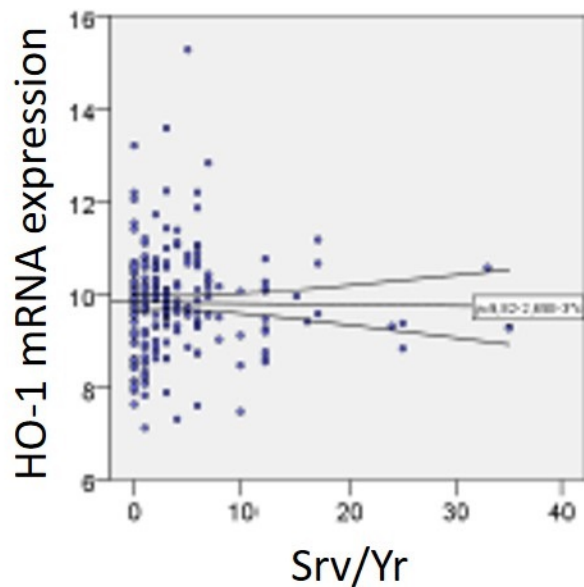

Figure S1: HO-1 mRNA expression and risk factors, tobacco and alcohol.

Supplement: Supplementary file 1 [file antioxidants-11-02077-s001.zip › antioxidants-1928406-supplementary.pdf]
